# Identification of *Staphylococcus aureus* Colony-Spreading Stimulatory Factors from Mammalian Serum

**DOI:** 10.1371/journal.pone.0097670

**Published:** 2014-05-15

**Authors:** Yosuke Omae, Kazuhisa Sekimizu, Chikara Kaito

**Affiliations:** Laboratory of Microbiology, Graduate School of Pharmaceutical Sciences, The University of Tokyo, Hongo, Bunkyo-ku, Tokyo, Japan; University Medical Center Utrecht, Netherlands

## Abstract

*Staphylococcus aureus* forms giant colonies on soft-agar surfaces, which is called colony-spreading. In the present study, we searched for host factors that influence *S. aureus* colony-spreading activity. The addition of calf serum, porcine serum, or silkworm hemolymph to soft-agar medium stimulated *S. aureus* colony-spreading activity. Gel filtration column chromatography of calf serum produced a high molecular weight fraction and a low molecular weight fraction, both of which exhibited colony-spreading stimulatory activity. In the low molecular weight fraction, we identified the stimulatory factor as bovine serum albumin. The stimulatory fraction in the high molecular weight fraction was identified as high-density lipoprotein (HDL) particles. Delipidation of HDL abolished the stimulatory activity of HDL. Phosphatidylcholine, which is the major lipid component in HDL particles, stimulated the colony-spreading activity. Other phosphatidylcholine-containing lipoprotein particles, low-density lipoprotein and very low-density lipoprotein, also showed colony-spreading stimulatory activity. These findings suggest that *S. aureus* colony-spreading activity is stimulated by albumin and lipoprotein particles in mammalian serum.

## Introduction

*Staphylococcus aureus* is a pathogenic bacterium that causes various diseases in humans, such as skin infections and sepsis. Methicillin-resistant *S. aureus* (MRSA) emerged in the 1960s and has caused serious clinical problems due to its multi-drug resistance. Hospital-associated MRSA (HA-MRSA) infects inpatients with weakened immune systems. Community-acquired MRSA (CA-MRSA) has recently become problematic due to its infection of healthy persons [1], [2], and is thought to have higher virulence than HA-MRSA. Understanding the molecular mechanisms of the virulence properties of this pathogen is important for developing therapeutic strategies against *S. aureus* infections.

We previously reported that *S. aureus* forms giant colonies on soft-agar surfaces at a speed of 1-cm in diameter/h [3]. We called this phenomenon “colony-spreading”. CA-MRSA strains have greater colony-spreading ability than HA-MRSA strains [4]. This difference in colony-spreading ability is attributed to the *psm-mec* gene locating in the cassette chromosome SCC*mec* [5], [6]. Introduction of the *psm-mec* gene into *S. aureus* suppresses its colony-spreading activity and virulence in mice [5]. Colony-spreading activity is correlated with *S. aureus* virulence. The recent finding of *S. aureus* colony-spreading activity on fresh pork meat [7] indicates a possible contribution of the host environment to *S. aureus* colony-spreading activity. The colony-spreading activity of *S. aureus* is inhibited by the self-secretion of delta-hemolysin [8] and cell-wall anchored proteins [9]. In contrast, colony-spreading requires the *agr* locus, which regulates exotoxin production [10]; *psmα*, which encodes cytolytic toxins [5]; wall teichoic acids [3]; and extracellular nucleases [11]. Thus, *S. aureus* components positively and negatively regulate its colony-spreading activity. Whether colony-spreading activity is also influenced by host factors, however, is not known.

In the present study, we assessed the effect of mammalian serum on *S. aureus* colony-spreading activity. Mammalian serum stimulated colony-spreading activity, and colony-spreading stimulatory molecules were identified.

## Results

### Serum stimulates *S. aureus* colony-spreading activity

**Figure 1:**
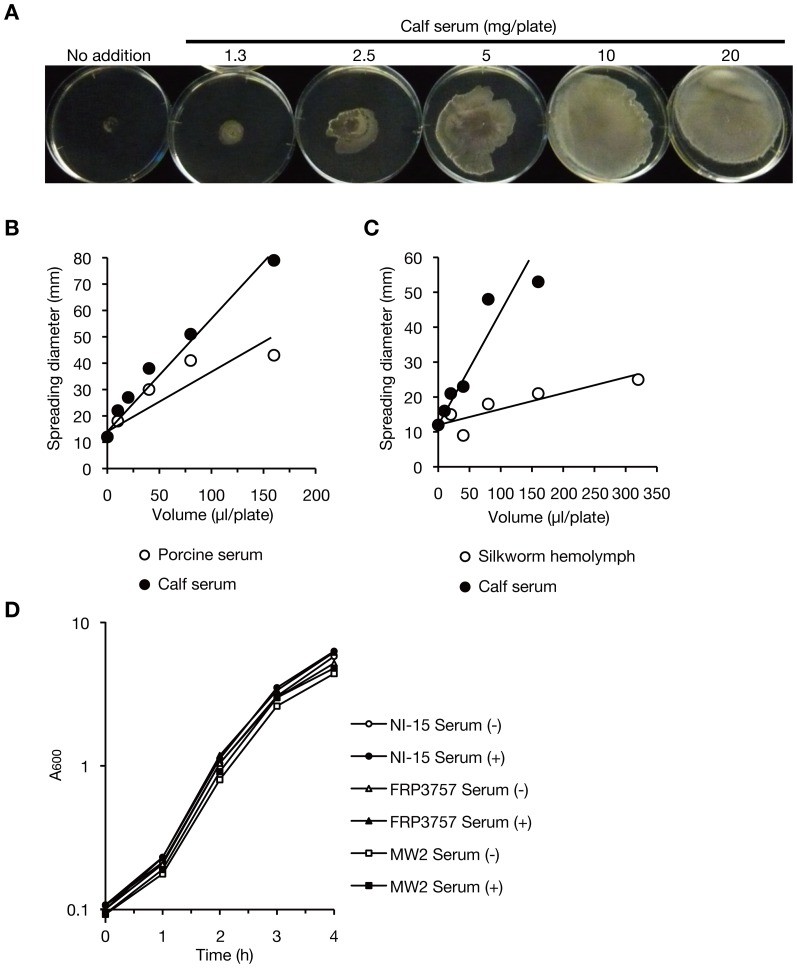
Mammalian serum stimulates *S. aureus* colony-spreading activity. (A) Stimulation of colony-spreading activity by calf serum. Overnight culture of *S. aureus* MRSA NI-15 was spotted on soft agar supplemented with serially diluted calf serum and incubated for 8 h at 37°C. Each plate contained 20 ml soft agar medium. (B) Stimulation of colony-spreading by porcine serum. Porcine serum or calf serum was serially diluted 2-fold and applied to 20 ml soft agar medium and its colony-spreading stimulatory activity was measured. Open circles indicate the halo diameters of giant colonies supplemented with porcine serum and filled circles indicate those supplemented with calf serum. Horizontal axis represents the volume of serum added to 20 ml soft agar medium in a plate. (C) Stimulation of colony-spreading activity by silkworm hemolymph. Hemolymph was collected from fifth instar larvae of silkworms and applied to the soft agar plates in 2-fold serial dilutions and its colony-spreading stimulatory activity was measured. Open circles indicate the halo diameters of giant colonies supplemented with silkworm hemolymph and filled circles indicate those supplemented with calf serum. (D) Growth curves of MRSA NI-15, MW2, or FRP3757 in tryptic soy broth supplemented with or without 1.25% (v/v) calf serum.

To evaluate the effect of host factors on colony-spreading, we examined whether the addition of mammalian serum changes the colony-spreading activity of *S. aureus* on soft agar. When soft agar medium was supplemented with calf serum, colony-spreading activity increased (Figure 1A). The stimulatory effect of calf serum increased in a dose-dependent manner (Figure 1B). We defined 1 unit of colony-spreading stimulatory activity as the fraction that stimulates colony-spreading 1.5-fold in 20 ml of soft agar medium and the stimulatory activity in calf serum was calculated to be 140 units/ml, or 2.1 units/mg protein. We further evaluated whether other types of host blood fluid had stimulatory activity, and found that both porcine serum and silkworm hemolymph stimulated colony-spreading (Figure 1B, 1C) with stimulatory activities of 100 units/ml for porcine serum and 40 units/ml for silkworm hemolymph. To determine whether the stimulatory activity of calf serum on colony-spreading is caused by the stimulation of bacterial growth, we examined whether the addition of serum affected *S. aureus* cell growth. The addition of 62.5 µl of calf serum to 5 ml of liquid medium, which corresponds to 250 µl of calf serum in a soft agar plate, did not stimulate *S. aureus* growth in liquid medium (Figure 1D), indicating that the stimulatory effects of serum on colony-spreading activity were not due to the effects of serum on bacterial growth.

### Diverse colony-spreading responses to calf serum among *S. aureus* strains

**Figure 2:**
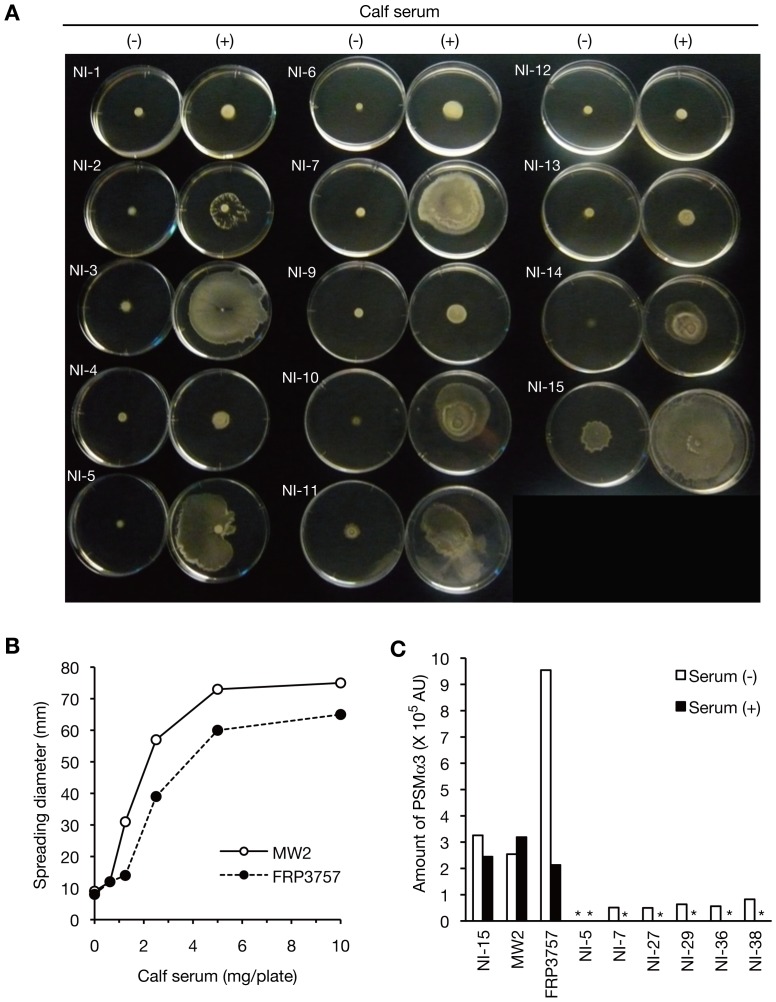
Diverse colony-spreading response to calf serum among HA-MRSA and CA-MRSA strains. (A) Representative images of the colony-spreading activity of MRSA NI strains stimulated by calf serum. Overnight cultures of MRSA NI strains were spotted onto soft agar plates supplemented with or without calf serum (250 µl/plate) and incubated for 8 h. (B) Colony-spreading response of CA-MRSA strains to calf serum. Overnight cultures of MW2 (USA400, open circles) or FRP3757 (USA300, filled circles) were spotted onto soft agar plates supplemented with 2-fold serially diluted calf serum and incubated for 8 h. The halo diameter was measured. (C) Amount of PSMα3 in MRSA strains cultured in the presence or absence of calf serum. MRSA strains with high colony-spreading response against calf serum (NI-15, MW2, FRP3757, NI-5, NI-7, NI-27, NI-29, NI-36, and NI-38) were cultured in the presence or absence of 1.25% (v/v) calf serum and the culture supernatants were analyzed by HPLC as described previously [6]. Asterisks means not detected.

We examined whether various *S. aureus* strains responded differentially to calf serum. Various *S. aureus* strains exhibited different colony-spreading responses to calf serum: 85% of the clinically isolated MRSA strains were stimulated less than 5-fold and 15% of strains were stimulated greater than 6-fold (Table 1, Figure 2A). Calf serum increased the colony-spreading activity of CA-MRSA strains FRP3757 and MW2 greater than 6-fold (Figure 2B). The addition of calf serum, however, did not stimulate growth of FRP3757 and MW2 (Figure 1D). These findings suggest that there are genetic variations among *S. aureus* strains that determine the colony-spreading response to serum. PSM-alpha protein is required for colony-spreading activity [5] and is highly expressed in CA-MRSA strains [12]. The amount of PSM-alpha, however, was not increased by the addition of calf serum (Figure 2C). This finding suggests that colony-spreading stimulation by serum is not due to increased expression of PSM-alpha.

**Table 1:** Colony-spreading responses of MRSA strains to calf serum.

with serum/without serum	Number of strains	*psm-mec* (+)	*psm-mec* (−)
×1.0–1.9	10	10	0
×2.0–2.9	12	9	3
×3.0–3.9	9	3	6
×4.0–4.9	3	1	2
×5.0–5.9	0	0	0
×6.0–6.9	3	3	0
×7.0–7.9	2	2	0
×8.0–8.9	1	1	0

Overnight cultures of MRSA NI strains (40 strains) [4] were spotted onto soft agar plates supplemented with or without calf serum (250 µl) and incubated for 8 h. Diameter ratios between plates with serum and plates without serum calculated for each strain are listed. The presence of the wild-type *psm-mec* gene was examined by sequencing.

### Serum albumin stimulates *S. aureus* colony-spreading

**Figure 3:**
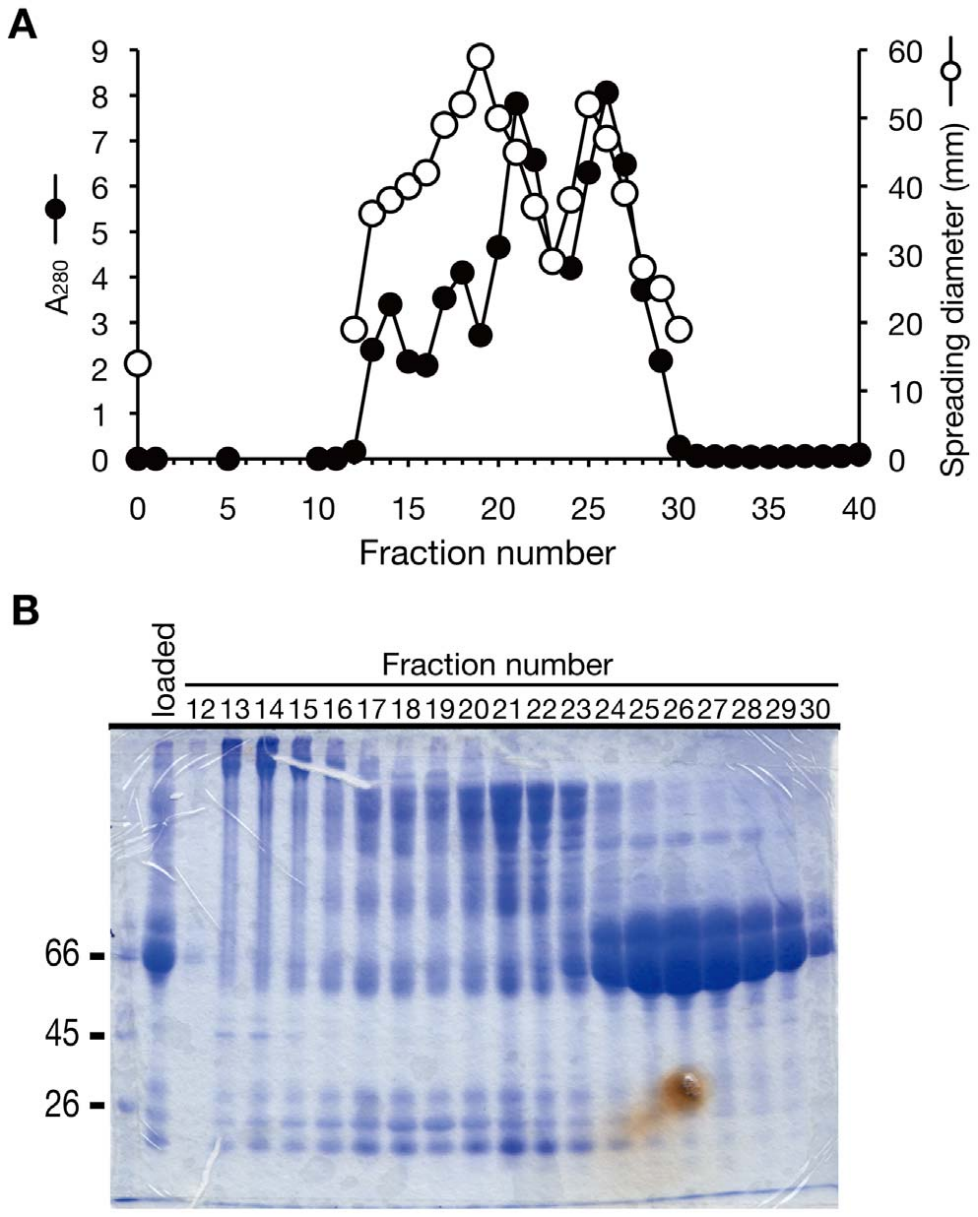
Gel filtration column chromatography of calf serum. (A) Elution profile of gel filtration column chromatography using a Superdex 200. Open circles indicate diameters of colonies. Filled circles indicate absorbance at 280 nm. Molecular weight markers were eluted in the fraction described below. Catalase (250-kDa) was eluted in fraction 21, bovine serum albumin (66-kDa) in fractions 25–26, and cyanocobalamin (1.3-kDa) in fraction 38. 250-µl aliquots of each sample were applied to soft agar medium and their colony-spreading stimulatory activity was measured. (B) SDS-PAGE analysis of gel filtration fractions. The gel was stained with Coomassie Brilliant Blue. A 66-kDa protein coincided with the colony-spreading stimulatory activity in fractions 24–29.

**Figure 4:**
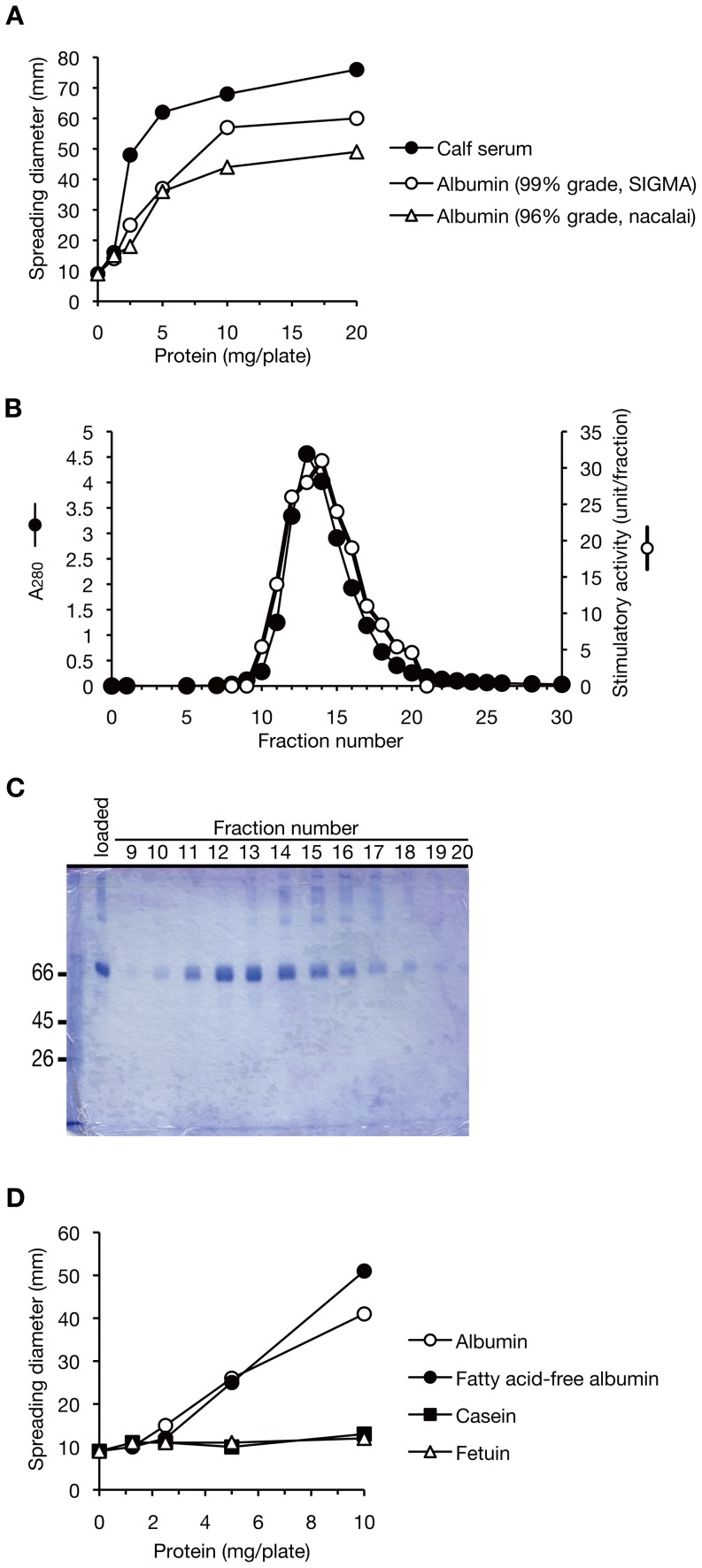
Stimulation of colony-spreading by bovine serum albumin. (A) Colony-spreading stimulation by purified bovine serum albumin. Purified bovine serum albumin of 96% grade (open triangles, Nacalai, cat. no. 01861-26) or 99% grade (open circles, Sigma, cat. no. A0281) was applied to soft agar by 2-fold serial dilution and their colony-spreading stimulatory activities against MRSA NI-15 strain were measured. (B) Elution profile of DEAE-cellulose column chromatography using bovine serum albumin (96% grade, Nacalai). Open circles indicate the colony-spreading stimulatory activity. Filled circles indicate absorbance at 280 nm. (C) SDS-PAGE analysis of DEAE-cellulose column chromatography fractions. The gel was stained with Coomassie Brilliant Blue. The 66-kDa protein coincided with colony-spreading stimulatory activity in fractions 10–20. (D) Bovine serum albumin (Nacalai), fatty acid-free albumin from bovine serum (Wako Chemicals, cat. no. 017-15146), casein from bovine milk (Sigma, cat. no. C4032), or fetuin from calf serum (Sigma, cat. no. F2379) was applied to soft agar by 2-fold serial dilution and their colony-spreading stimulatory activities on the MRSA NI-15 strain were measured.

To reveal the molecular mechanism of the colony-spreading stimulation by serum, we purified the stimulatory molecule from calf serum. Gel-filtration column chromatography was used to separate the colony-spreading stimulatory activity into two fractions, a high molecular weight fraction and a low molecular weight fraction (Figure 3A). The findings suggested the presence of at least two stimulatory molecules in calf serum. According to the molecular weight markers in the gel-filtration column chromatography, the molecular mass of the stimulatory factor with a low molecular weight was 66 kDa, and analysis by sodium dodecyl sulfate-polyacrylamide gel electrophoresis (SDS-PAGE) revealed that the major component of the fraction was a 66-kDa protein (Figure 3B). Serum albumin is the major component of serum and its molecular weight is 66 kDa. We used purified bovine serum albumin to examine whether serum albumin has colony-spreading stimulatory activity. Purified albumin stimulated colony-spreading activity (Figure 4A). Because the purified albumin fraction contained other high molecular weight proteins, we performed ion-exchange column chromatography to examine the coincidence of albumin with colony-spreading activity. In DEAE cellulose column chromatography, the stimulation of colony-spreading activity indeed coincided with the presence of albumin and did not coincide with the presence of several faint high molecular weight proteins (Figure 4B, 4C). As fatty acids associate with albumin in serum, we next examined whether fatty acid-free albumin enhanced colony-spreading activity. Fatty acid-free albumin stimulated colony-spreading (Figure 4D). These findings indicate that albumin proteins act to stimulate colony-spreading. Serum albumin is the most abundant protein in animal serum, and thus it is possible that any abundant protein could enhance colony-spreading activity. We therefore examined the specificity of the serum albumin-induced colony-spreading. The addition of casein, a milk protein, or fetuin, a blood protein, did not stimulate colony-spreading activity (Figure 4D), indicating that serum albumin specifically stimulates colony spreading. The stimulatory activity of albumin was 0.9 unit/mg protein, which was lower than that of calf serum (2.1 unit/mg protein). Because albumin accounts for 50% to 65% of the whole proteins of calf serum, albumin explains 21 to 28% of the total stimulatory activity induced by calf serum. In addition, albumin was not detected in the active fractions with a high molecular weight in gel-filtration chromatography (Figure 3B). These findings suggest the presence of an additional stimulatory factor in calf serum.

### Purification of the colony-spreading stimulatory factor in calf serum

**Figure 5:**
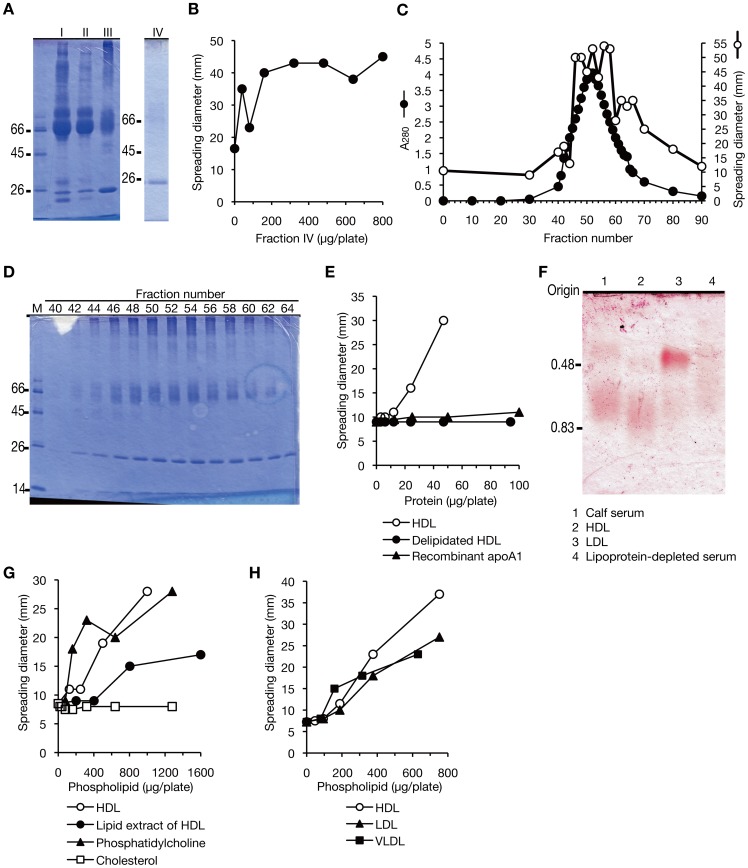
Identification of lipoprotein particles as a colony-spreading stimulator. (A) Protein from each purification step was electrophoresed by SDS-PAGE. The gel was stained with Coomassie Brilliant Blue. (B) The final fraction (Fraction IV) was serially diluted 2-fold, and the colony-spreading stimulatory activity was measured. (C) Elution profile of DEAE-cellulose column chromatography using Fraction III. *Open circles* indicate diameters of colony-spreading. *Filled circles* indicate absorbance at 280 nm. 500-µl aliquots of each sample were applied to the soft agar medium and their colony-spreading stimulatory activity was measured. (D) SDS-PAGE analysis of DEAE-cellulose column chromatography fractions. The 25-kDa protein coincided with colony-spreading stimulatory activity in fractions 46-58. The 25-kDa protein was identified as apolipoprotein A1 by peptide-mass fingerprinting. (E) Colony-spreading stimulation by HDL particles. Purified HDL, delipidated HDL, or recombinant human apolipoprotein A1 (Wako Chemicals, cat. no. 019-20731) was applied to soft agar by 2-fold serial dilution and their colony-spreading stimulatory activities on the MRSA NI-15 strain were measured. Human apolipoprotein A1 shares 78% amino acids identity with calf apolipoprotein A1. (F) Agarose gel electrophoresis analysis of calf serum, HDL, LDL, and lipoprotein-depleted serum. Agarose gel electrophoresis was performed according to a previous report [23]. Each fraction (4 µg phospholipid) was electrophoresed in agarose 0.5%, and then lipoprotein bands were detected by lipid-specific staining using Oil Red O. The electrophoresed protein amount of calf serum, HDL, LDL, and lipoprotein-depleted serum was 500 µg, 5 µg, 5 µg, and 1800 µg, respectively. (G) Colony-spreading stimulation by phosphatidylcholine. Purified HDL, lipid extract from HDL, phosphatidylcholine from egg yolk (99% purity, Sigma, cat. no. P3556), or cholesterol (99% purity, Sigma, cat. no. C8667) was applied to soft agar by 2-fold serial dilution and their colony-spreading stimulatory activities on the MRSA NI-15 strain were measured. (H) Colony-spreading stimulation by HDL, LDL, and VLDL. Fractionated HDL, LDL, or VLDL was applied to soft agar by 2-fold serial dilution and their colony-spreading stimulatory activities on the MRSA NI-15 strain were measured.

To identify the colony-spreading stimulatory molecule other than albumin, we purified the stimulatory molecule from calf serum by measuring the increase in specific activity to stimulate colony-spreading. The specific activity was increased 45-fold by precipitation with ammonium sulfate, boiling, and butyl-Toyopearl column chromatography (Table 2). SDS-PAGE analysis of the final fraction (Fraction IV) revealed that the fraction contained a major 25-kDa protein (Figure 5A). The total recovery of activity in Fraction IV was 10% and its specific activity was 25 units/mg protein (Figure 5B, Table 2). The 25-kDa protein was present in the high molecular weight fraction in gel-filtration column chromatography (Figure 3B), and coincided with the colony-spreading stimulatory activity in DEAE-cellulose column chromatography (Figure 5C, 5D). Peptide-mass fingerprinting revealed that the 25-kDa protein was apolipoprotein A1. Apolipoprotein A1, whose theoretical molecular weight is 28 kDa, migrates on SDS-PAGE at 25 kDa [13]. Because Fraction IV contained traces of proteins other than the 25-kDa protein, we examined whether recombinant human apolipoprotein A1 stimulates colony-spreading. The recombinant apolipoprotein A1 did not stimulate colony-spreading (Figure 5E), indicating that other factors are required for the stimulatory activity. Apolipoprotein A1 is a major component of high-density lipoprotein (HDL) and elutes in the high molecular weight fraction when serum is separated by gel filtration column chromatography [14]. To determine whether HDL particles stimulate colony-spreading activity, we isolated HDL particles by high-density ultracentrifugation (Figure 5F) and assessed the stimulatory activity. HDL had colony-spreading stimulatory activity (Figure 5E), and recovery of the activity from serum was 41%. To determine whether the stimulatory activity of HDL was due to the protein or lipid component of HDL, HDL was delipidated. HDL proteins that were delipidated by treatment with diethyl ether and methanol lost their stimulatory activity (Figure 5E). In contrast, a lipid extract of HDL stimulated colony-spreading activity (Figure 5G). To further identify the lipid component responsible for the stimulatory activity, we analyzed the effect of the major lipid components of HDL, cholesterol and phosphatidylcholine. Phosphatidylcholine, but not cholesterol, had a colony-spreading stimulatory effect (Figure 5G). Taking the percentage of phosphatidylcholine (75 wt% in HDL phospholipids) [15] into consideration, the presence of phosphatidylcholine could explain the total stimulatory activity of HDL (Figure 5G). These findings suggest that HDL particles containing phosphatidylcholine in serum stimulate *S. aureus* colony-spreading activity. Other lipoproteins besides HDL contain phosphatidylcholine. We assessed the effect of other lipoprotein particles, low-density lipoprotein (LDL) and very low-density lipoprotein (VLDL), which are reported to contain phosphatidylcholine [15]. Like HDL, the addition of LDL or VLDL to soft-agar medium stimulated colony-spreading (Figure 5H). The recovery of activity from serum attributed to LDL and VLDL was 2.7% and 0.6% each, more than 10-times lower than that accounted for by HDL. These findings suggest that lipoprotein particles containing phosphatidylcholine stimulate colony-spreading activity.

**Table 2:** Purification of colony-spreading stimulatory factor in calf serum.

Fraction		Volume (ml)	Total activity (Unit)	Yield (%)	Protein (mg)	Specific activity (Unit/mg)
I	Calf serum	19	880	100	1600	0.55
II	Ammonium sulfate precipitate	27	1060	120	1080	0.98
III	Heat treated supernatant	20	330	38	60	5.5
IV	Butyl-Toyopearl	4.5	90	10	4	25

Calf serum (19 ml) was used as the starting medium. To measure the colony-spreading stimulatory activity, samples were serially diluted 2-fold and applied to 20 ml of soft agar and incubated for 8 h at 37°C. The diameters of giant colonies were measured from each dish, and stimulatory activities were calculated.

### Depletion of lipoprotein particles and albumin decreases the stimulatory activity of serum

**Figure 6:**
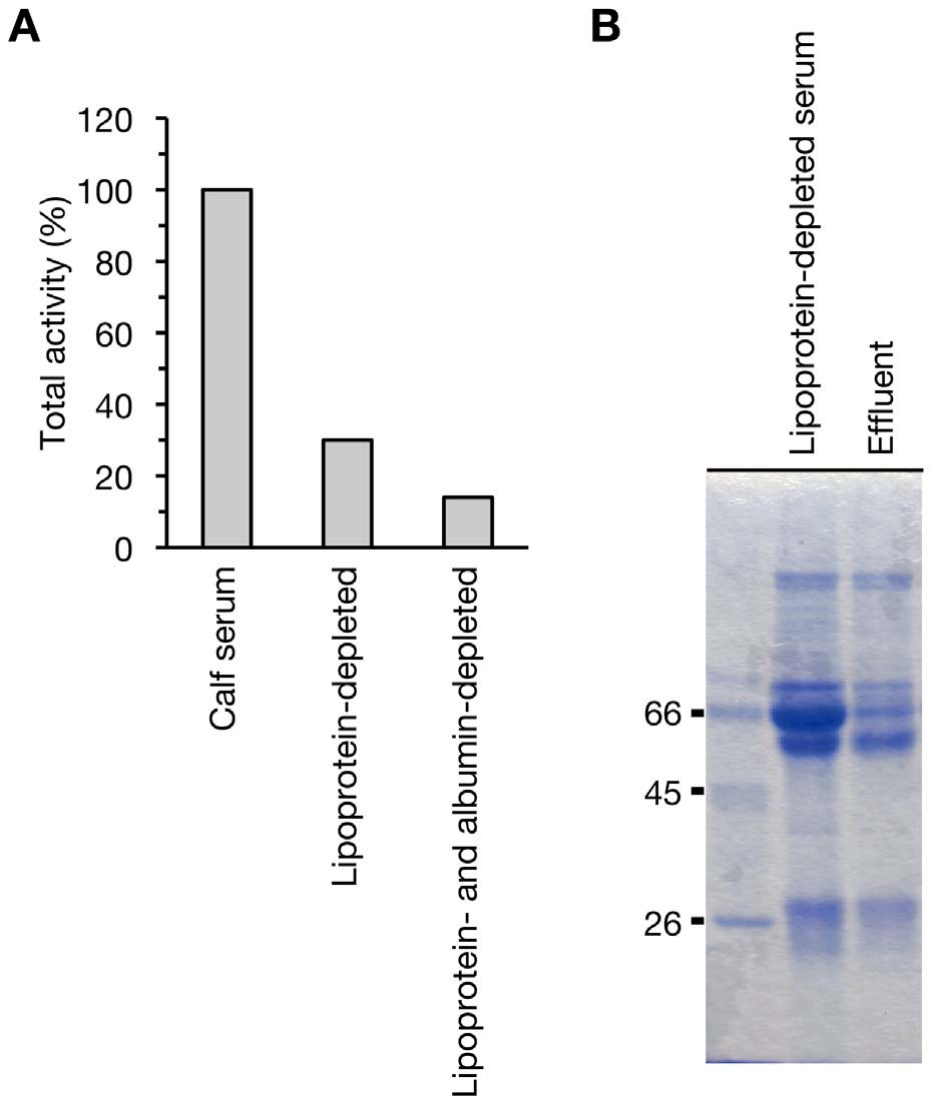
Lipoprotein and albumin-depleted serum decreases colony-spreading stimulatory activity. (A) Stimulatory activities of calf serum, lipoprotein-depleted serum, lipoprotein- and albumin-depleted serum were examined. Fractionation was performed from a 50-ml volume of calf serum containing 3850 units of stimulatory activity. Each fraction was applied to soft agar by 2-fold serial dilution and their colony-spreading stimulatory activities on the MRSA NI-15 strain were measured. Relative total activities of each fraction compared with that of calf serum are presented in graph. (B) SDS-PAGE analysis of the lipoprotein-depleted serum and Affi-Gel blue gel column effluent. 10 µg protein of the lipoprotein-depleted serum and the Affi-Gel blue gel column effluent was electrophoresed in 12.5% SDS-polyacrylamide gel.

To confirm the contribution of lipoproteins and albumin to the total activity of serum to stimulate colony-spreading, we depleted the lipoprotein particles and albumin from serum and examined the effect on the stimulatory activity. Lipoprotein particles were depleted by high-density ultracentrifugation, resulting in lipoprotein-depleted calf serum containing 28% of phospholipids. Calf serum and the lipoprotein-depleted serum containing the same amount of phospholipids were electrophoresed and the decrease of LDL and HDL in the lipoprotein-depleted serum was confirmed (Figure 5F). The stimulatory activity of the lipoprotein-depleted serum was reduced to 30% (Figure 6A). We further removed albumin from the lipoprotein-depleted serum by Affi-Gel blue gel column chromatography, in which albumin protein is absorbed to Cibacron Blue F3GA dye. The amount of albumin protein was decreased (Figure 6B) and the total activity of the fraction decreased to 14% (Figure 6A). These findings suggest that albumin and lipoprotein particles are responsible for over 80% of the stimulatory activity of serum, and other unidentified colony-spreading stimulatory factor(s) are present in serum.

## Discussion

The findings of the present study demonstrated that albumin and lipoprotein particles in calf serum stimulate *S. aureus* colony-spreading activity. This study is the first to identify host factors that stimulate *S. aureus* colony-spreading activity. Because these factors are abundant in host serum, it is assumed that *S. aureus* colony-spreading is facilitated in the host environment. One possibility is that albumin and lipoprotein particles exuded from the blood following injury facilitate *S. aureus* colony-spreading activity in the wet wound site. We also revealed that phosphatidylcholine in lipoprotein particles stimulates the colony-spreading behavior. Phosphatidylcholine is abundant on the lung surfaces [16], [17], and thus *S. aureus* colony-spreading activity might be enhanced on the alveolar surfaces of the lung. *S. aureus* colony-spreading in such host environments should be further investigated.

We also revealed that various *S. aureus* clinical isolates respond differentially to stimulation by calf serum, suggesting that the molecular mechanisms in *S. aureus* involved in the colony-spreading response to host factors differ among *S. aureus* strains. The presence of the *psm-mec* gene did not correlate with the colony-spreading response to serum among clinically isolated MRSA strains (Table 1). The expression of PSM-alpha protein, which is both required for colony-spreading and downregulated by *psm-mec*, also did not correlate with the high colony-spreading response to serum (Figure 2C). Thus, the colony-spreading response to serum is not strictly dependent on *psm-mec* and is not due to the altered expression of PSM-alpha. The colony-spreading activity of CA-MRSA strains, which are virulent to healthy individuals, was highly stimulated by serum, suggesting that high responsiveness to serum contributes to the high virulence of CA-MRSA strains. Further studies are needed to identify the genetic determinant of the colony-spreading responsiveness to serum and to reveal the correlation between the serum responsiveness of a strain and its clinical effects.

We did not reveal the molecular mechanisms underlying the stimulation of colony-spreading by albumin and phosphatidylcholine in lipoprotein particles in the present study. Both serum albumin and phosphatidylcholine adsorb biologic molecules. Serum albumin adsorbs free fatty acids and lipid soluble hormones [18], and phosphatidylcholine binds lipids. Some of their adsorptions are mediated by hydrophobic interactions [19], [20]. *S. aureus* secretes delta-hemolysin, which is a hydrophobic inhibitor of colony-spreading [8]. Extracellular DNA, which is secreted by dead bacterial cells, inhibits colony-spreading activity [11]. Adsorption of these inhibitory molecules by serum albumin or phosphatidylcholine might be a mechanism to stimulate *S. aureus* colony-spreading activity. HDL indeed has the capacity to bind phenol soluble modulins [21]. In addition, serum albumin and phosphatidylcholine might affect gene expression in *S. aureus*, thereby stimulating colony-spreading. Phosphatidylcholine has a surfactant property [16], which might dynamically facilitate *S. aureus* colony-spreading on wet surfaces. Further experiments are needed to examine these possibilities.

## Materials and Methods

### Mammalian serum

Calf serum was purchased from Equitech-Bio, Inc. (Kerrville, TX). Porcine serum was purchased from Nippon Biotest Laboratories Inc. (Tokyo, Japan).

### Spreading stimulation assay

Colony-spreading studies were performed using the MRSA NI-15 strain with type IV SCC*mec*. An overnight culture (2 µl) was spotted onto the soft-agar plate, dried for 15 min, and incubated at 37°C for 8 h. Samples were added to 20 ml soft-agar plates and poured into 80-mm dishes.

### Growth experiment in the presence of serum

Overnight cultures of *S. aureus* strains were 100-times diluted with fresh tryptic soy broth medium supplemented with or without 1.25% (v/v) calf serum and aerobically cultured at 37°C. Their absorbance at 600 nm (A600) was measured using a spectrophotometer (UV-1200, Shimadzu).

### Calf serum sample preparation

Ammonium sulfate was added to calf serum to obtain a 33% (w/v) solution, centrifuged at 15000 *g* for 30 min, and supernatant was collected. The supernatant was then subjected to 100% ammonium sulfate precipitation and centrifuged at 15000 *g* for 30 min. The pellet was resuspended in buffer A (50 mM Tris-HCl buffer [pH 8.0]) and dialyzed with buffer A (Fraction II). Fraction II was heat-treated at 100°C for 10 min, and the supernatant was collected by ultracentrifugation (50.2Ti rotor, 302,000 *g*, 1 h; Fraction III). The butyl-Toyopearl column was equilibrated with buffer A. Fraction III (20-ml) was loaded onto the butyl-Toyopearl (1.5×3 cm), washed with buffer A, and eluted with buffer A containing 50% EtOH (5 ml/fraction). The elution fractions were pooled and dialyzed with buffer A, loaded onto a DEAE-cellulose column (1.5×3 cm), eluted with buffer A containing 0.5 M NaCl, and dialyzed with buffer A (Fraction IV). The protein concentration was determined by the Bradford method using bovine serum albumin as the standard. Protein determination was performed by matrix-assisted laser desorption-ionization time-of-flight mass spectrometry analysis (GENOMINE, INC., South Korea) using trypsin digestion. The protein was identified as apolipoprotein A-1 using a MASCOT Search with sequence coverage of 79%.

### Gel filtration column chromatography

Filtered calf serum (500 µl) was loaded on a Superdex 200 (GE Healthcare), and fractionated (0.5 ml/fraction).

### DEAE-cellulose column chromatography of albumin

Albumin (Nacalai) was suspended in buffer A, precipitated with ammonium sulfate (15,000 *g*, 10 min), resuspended in buffer A, and dialyzed with buffer A. The dialysate was loaded onto the DEAE-cellulose column (1.5×15 cm), washed with buffer A, and eluted with 0–0.5 M NaCl gradient (8 ml/fraction).

### Isolation of serum HDL particles by high-density ultracentrifugation

High-density lipoproteins were isolated according to a previous report [22]. Briefly, calf serum was centrifuged for 45 min at 48,300 *g*. Serum-free chylomicrons were then centrifuged for 24 h at 302,000 *g*. The upper phase containing very low-density lipoproteins was obtained as the VLDL fraction. The lower phase was adjusted to a density of 1.063 g/ml by the addition of solid NaBr and centrifuged for 24 h at 302,000 *g*. The upper phase containing low-density lipoproteins was obtained as the LDL fraction. The lower phase was adjusted to a density of 1.23 g/ml by the addition of solid NaBr and centrifuged for 60 h at 245,000 *g*. High-density lipoproteins were obtained from the upper phase while the lower phase was used as the lipoprotein-depleted serum. All ultracentrifugation was performed at 4°C using a Beckman 50.2 Ti rotor (Beckman Instrument). The recovered lipoprotein fractions were then dialyzed against buffer A and the phospholipid concentration was determined using a phospholipid kit (Wako Chemicals).

### Delipidation of HDL

The lipid extraction of HDL was performed as described previously [22]. Briefly, 1 volume of HDL isolated by ultracentrifugation was slowly added to 12 volumes of ice-cold methanol with constant stirring. Then, 28 volumes of ice-cold diethyl ether were added to the solution. After 10 min stirring on ice, the mixture was centrifuged at 500 *g* for 5 min. The protein pellet was resuspended in 40 volumes of diethyl ether. After 10 min stirring on ice, the mixture was centrifuged as above. The pellet was recovered and dried. To minimize aggregation, the delipidated HDL lipoproteins were solubilized at 2 mg/ml protein in 50 mM Tris-HCl (pH 8.0) containing 100 mM NaCl, 1 mM EDTA, and 2 M guanidine hydrochloride and then dialyzed against buffer A. The methanol/diethyl ether extract was evaporated, dialyzed against buffer A, and used as the lipid fraction of HDL.

### Albumin removal by Affi-Gel column chromatography

Albumin removal was performed using Affi-Gel blue gel, 50–100 mesh (Bio-Rad) according to the manufacturer's protocol. Lipoprotein-depleted serum collected as described above was dialyzed against buffer A, and 100 mg of dialyzed sample was loaded onto the 10 ml Affi-Gel blue gel column equilibrated with buffer A. Its effluent recovered by the wash with 8 ml buffer A was used as the effluent fraction.
